# Near-infrared fluorescence guided laparoscopic cholecystectomy in the spectrum of complicated gallstone disease

**DOI:** 10.1097/MD.0000000000031170

**Published:** 2022-10-21

**Authors:** Srikanth Gadiyaram, Ravi Kiran Thota

**Affiliations:** a Department of Surgical Gastroenterology and Minimally Invasive Surgery, Sahasra Hospitals, Jayanagar, Bangalore, India.

**Keywords:** bilioenteric fistula, common bile duct stones, complicated gallstones, ICG fluorescence guidance, laparoscopic cholecystectomy

## Abstract

ICG fluorescence (ICGF) guidance during laparoscopic cholecystectomy (LC) is gaining wider acceptance. While the accruing data largely addresses ICGF guidance during LC in patients with uncomplicated gallstone disease (UGS) and acute cholecystitis, there is a paucity of data related for complicated gall stone disease (CGS) such as choledocholithiasis, bilio-enteric fistula, remnant gall bladder, etc. The purpose of this study was to evaluate the role of ICGF during LC in the spectrum of CGS with state of the art 4 chip camera system. Retrospective review from a prospectively maintained database of **all** patients who underwent ICGF guided LC during the period June 1^st^, 2019 till December 30^th^, 2021 formed part of the study. Clinical profile and findings on ICGF during LC for CGS were studied. The data was studied to evaluate the potential roles of ICGF during LC for CGS. Of 68 patients, there were 29 males and 39 females. Among them were 32 and 36 in the uncomplicated and complicated gallstone disease groups, respectively. ICGF showed CBD visualization in 67(98.5%) and cystic duct in 62(91%). ICGF guidance helped in management of CGS, prior to, during and after completion of LC. It had novel application in patients undergoing CBD exploration. In our small series of patients with CGS, ICGF guidance enabled a LC and laparoscopic subtotal cholecystectomy in 94% and 6% of patients respectively. The study highlights potential roles and advantages with ICGF guided laparoscopic management for CBD stones, bilioenteric fistula, completion cholecystectomy and cystic duct stones. Large scale multicenter prospective studies are required to clarify the role of ICGF in the wide spectrum of CGS.

## 1. Introduction

ICG fluorescence (ICGF) guidance during laparoscopic cholecystectomy (LC) is gaining wider acceptance and the recent Shanghai consensus statement on fluorescence guided LC is an additional evidence auguring its greater use in future.^[1–4]^ Gall stone disease has a wide spectrum of clinical presentation and can have unusual findings during the course of surgery. The accruing data largely addresses issues pertaining to ICGF guidance in uncomplicated gallstone disease (UGS) and in patients with acute cholecystitis. There is a paucity of data pertaining to role of ICGF during laparoscopic management of complicated gall stone disease (CGS). This is with particular reference to CGS other than acute cholecystitis such as choledocholithiasis, bilio-enteric fistula and in surgery for remnant gall bladder.

The risk of bile duct injury during LC is higher in patients with CGS. ICGF during LC would be particularly useful for identification of extra-hepatic biliary tree in these patients.^[5]^ In patients with acute cholecystitis a greater difficulty in visualizing the biliary tree with the use of ICGF secondary to inflamed and thickened tissues has been reported.^[6]^ Nevertheless, evolving ICG related laparoscopic technology could possibly overcome some of these challenges.

The present study evaluates the role of ICGF cholangiography during LC for CGS utilizing a state of the art laparoscopic near-infrared fluorescence (NIRF) imaging system with a “four chip” camera.

## 2. Materials and Methods

Data of all patients who underwent ICGF guided LC (by senior author SG) during the period June 1^st^, 2019 till December 30^th^, 2021 were reviewed retrospectively from a prospectively maintained database. The demographic profiles, indications, details of administration of ICG, findings at surgery, in these patients were studied. Patients were categorized into UGS and CGS disease groups. UGS group consisted of patients with biliary colic alone. The CGS group included patients with one or more of the following complications, viz; acute cholecystitis, CBD stones, and gallstone pancreatitis, bilio-enteric fistulae and remnant gallbladder stones. The data was studied to evaluate the potential roles of ICGF during LC for CGS. Since the study was retrospective in nature, an institutional review board approval was not obtained. An informed written consent was obtained from all patients who underwent ICGF guided LC.

### 2.1. Multispectral near-infrared imaging system

Maxer Endoscopy GmbH (Germany), VironX system with VironX NIR light was used for fluorescence guided surgery. The camera head is “four chip,” buttonless and intuitive. The presence of “red,” “blue,” “green,” and “NIR” sources enabled clear images and real time navigation with NIR overlay images in real time with natural color. Control of image on laparoscopic monitor can be done from a tablet (touchpad operated by the technician), keyboard or footswitch (operated by surgeon). On instruction by the operating surgeon, the operation room technician using the touchpad could provide “picture in picture” images of NIRF, regular laparoscopic image and near infrared fluorescence augmented (NIRFA) image, all in real-time. The fluorescence image is augmented in real time to white light image. All procedures were recorded with the VironX recording system with regular laparoscopic images, NIRF and NIRFA available for review. Details pertaining to visualization of fluorescence of extrahepatic biliary tree including fluorescence of common bile duct, cystic duct and cystic duct/common bile duct junction during NIR guidance were recorded. In patients with non-visualization of any or all of the above in the augmented image, the simultaneously acquired NIR images were studied (vide infra).

### 2.2. Indocyanine green for injection

AUROGREEN (Aurolab, Madurai, TN, India), 25 mg vials containing powder for reconstitution, 10 mL of sterile water/syringe provided in the package. Also, included in the package is a filter for use during intravenous administration of drug to filter out particulate matter.

### 2.3. Timing and dosing of ICG

All patients received 2.5 mg (1 mL), intravenously of ICG 2 hours prior to the procedure.

### 2.4. Operative technique

All procedures were done laparoscopically under ICGF guidance and. Dissection was done to achieve a critical view of safety (CVS). Laparoscopic D2 first approach was used in 4 patients for an obscure gall bladder described by senior author (SG) previously.^[7]^ Bailout strategies such as subtotal cholecystectomy were employed as required where deemed necessary. In patients with CBD stones a transcholedochal laparoscopic CBDE (LCBDE) was performed. Patients with other complications were addressed as required. Sub hepatic drains were placed selectively. An intraoperative cholangiogram (IOC) was performed on need basis (deranged liver function tests, as a part of common bile duct exploration and in patients with history of gall stone related pancreatitis).

### 2.5. Definitions

**Figure 1.**
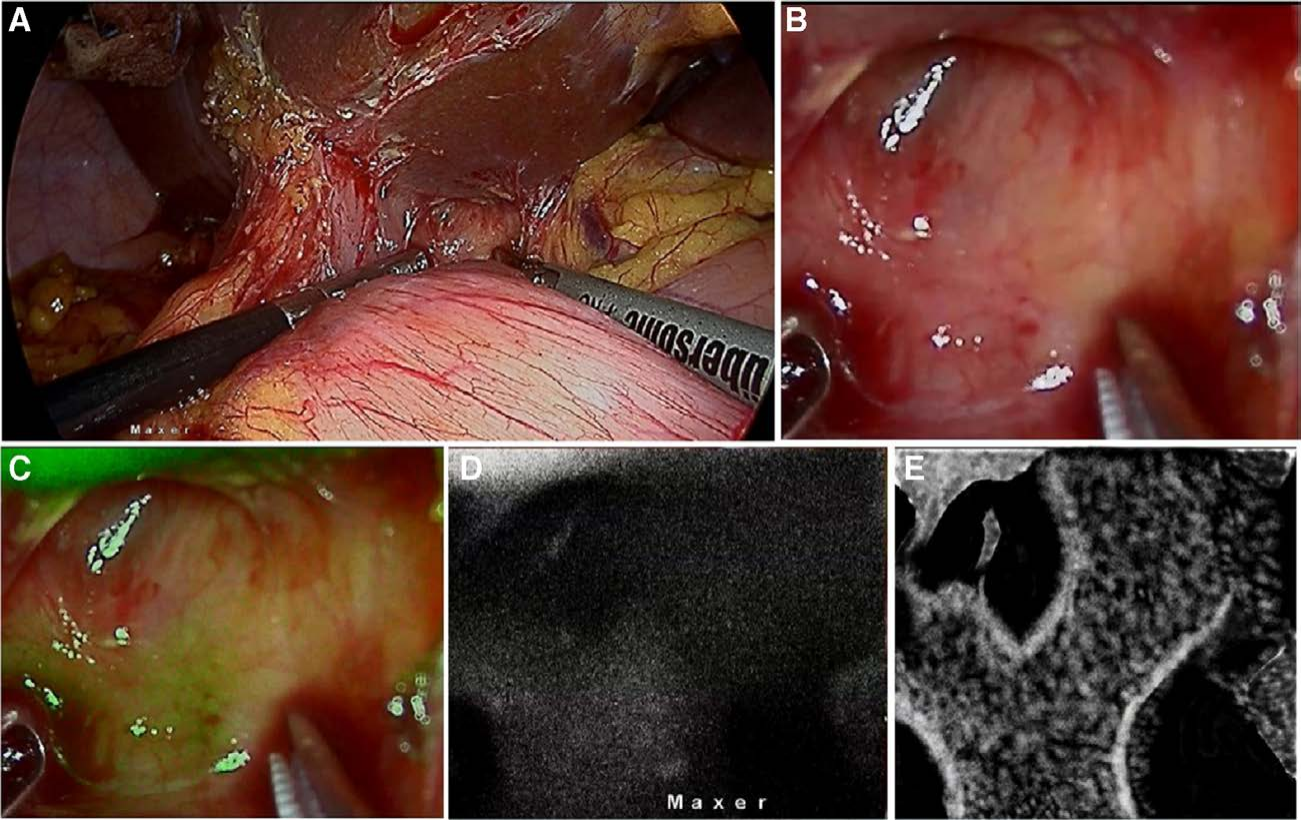
ICGF in a patient undergoing completion cholecystectomy. (a) Laparoscopic image in left upper corner showing a duodenal fistula to remnant and an obscure hepatocystic triangle; (b, c, d) Close up view of the hepatocystic triangle area in pure laparoscopy, NIRFA and NIRF modes respectively; E. Illustration of the NIRF image interpretation showing the location of common hepatic, CD and CBD. CBD = common bile duct, CD = cystic duct, ICGF = ICG fluorescence, NIRF = near-infrared fluorescence, NIRFA = near infrared fluorescence augmented.

NIRF—These are images acquired in the near infra-red mode which are typically seen as black background with fluorescence seen in “white.” This is the “true” near-infrared-fluorescence image (Fig. 1).

NIRFA—The computer processes the NIRF image along with the laparoscopic image and produces an overlay of the NIRF image onto the laparoscopic image. Typically, the color coding for biliary system is green. The camera being a 4 chip one, the true natural color of the laparoscopic image is preserved with only the biliary system showing up as “green.” Augmented images are thus computer generated to facilitate visual perception of operating surgeon.

Images—This word appears during the course of description of results, discussion (NIRF image, NIRFA image, laparoscopy image) and must be understood as “freeze” images or “grab images” from actual videos to enable easy understanding of findings and comparisons thereof. In reality all the recordings of NIRF or NIRFA or Laparoscopy were real time video captures with grab images taken from the tablet by the OT technician.

Real-time: The live video seen on the monitor as seen by the surgeon as a continuum during the conduct of the operation.

### 2.6. Statistical analysis

This was done with SPSS version 22 (IBM SPSS Statistics, Somers NY). Categorical data was represented in the form of Frequencies and proportions. Chi-square test or Fischer’s exact test (for 2 × 2 tables) was used as test of significance for qualitative data. “p” value of <.05 was considered as statistically significant.

## 3. Results

Sixty-eight patients, 29 males and 39 females formed part of the study group. The median age was 51 (range 20–92) years, BMI 24.5 (17.78–63.7) kg/m^2^ and 28 patients had one or more co-morbidities. Of them, 32 and 36 patients belonged to the UGS and CGS groups, respectively. The number of male:female patients were 12:20 and 17:19, and BMI was 24.7 (17.8–63.7)kg/m^2^ and 24.6 (19.8–28)kg/m^2^, in UGS and CGS groups, respectively. None of the patients had an allergy to iodine or sea food. None of the patients had cirrhosis.

Of the 36 patients in CGS group, there were 17 male and 19 female patients with a median BMI 24.6 (19.8–28) kg/m^2^. Twenty-four patients in CGS group belonged to acute cholecystitis group based on Tokyo guidelines (Grade I in 21 patients, grade II in 2 patients and Grade III in 1 patient).^[8]^ Of these, 2 patients had gangrenous cholecystitis (Tokyo grade II) who underwent an emergency LC. They received intravenous ICG, 2.5 mg 2 hours prior to surgery. Sixty-seven patients underwent traditional multiport LC while 1 patient underwent a single incision LC.

**Figure 2.**
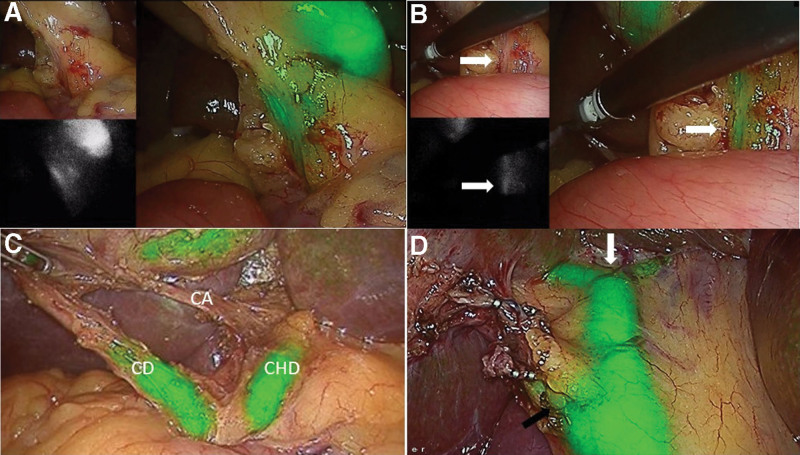
(a) ICGF at the beginning of dissection of hepatocystic triangle showing real time laparoscopic image in left upper corner, NIRF in left lower corner and NIRFA on right side; (b) Instrument passed from epigastric port representing the line drawn from falciform ligament to the right end of Rouviere’s sulcus showing the CBD below the instrument, white arrow showing CBD; (c) ICGF during demonstration of critical view of safety; (d) ICGF showing extrahepatic biliary tree at the conclusion of laparoscopic cholecystectomy, white arrow showing confluence of right and left hepatic ducts at hilum, black arrow showing cystic duct and CBD junction. CBD = common bile duct, ICGF = ICG fluorescence, NIRF = near-infrared fluorescence, NIRFA = near infrared fluorescence augmented.

**Figure 3.**
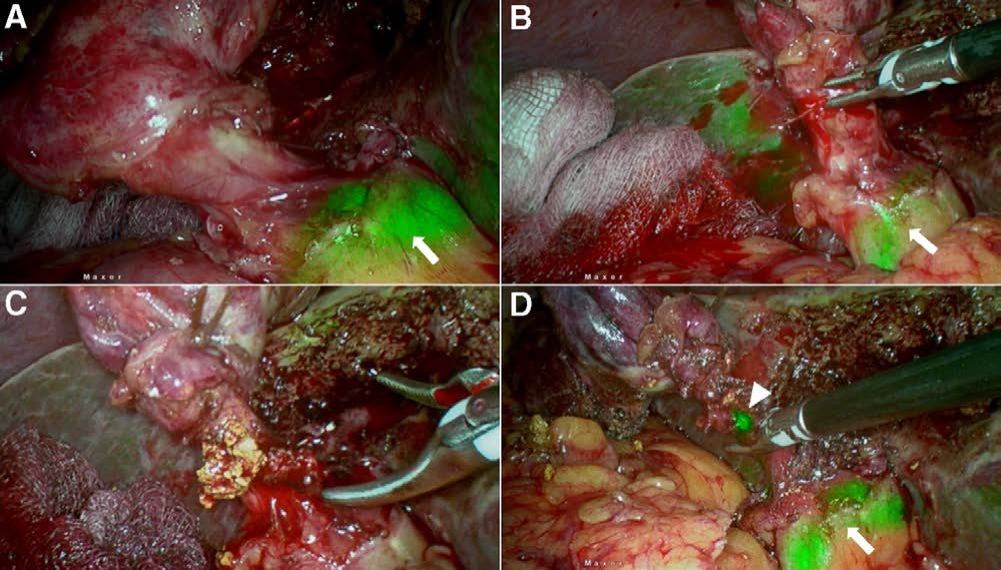
(a) ICGF after antegrade dissection of gallbladder, arrow showing CBD fluorescence, showing a wide cystic duct with suspicion of cystic duct calculi; (b) Bleeder from cystic ductotomy, arrow showing CBD fluorescence, CBD fluorescence not obscured with blood in field; (c) Stone milked out from cystic duct; (d) Arrow head showing fluorescent bile leak from ductotomy site after cystic ductstone clearance, arrow showing CBD fluorescence. CBD = common bile duct, ICGF = ICG fluorescence.

**Figure 4.**
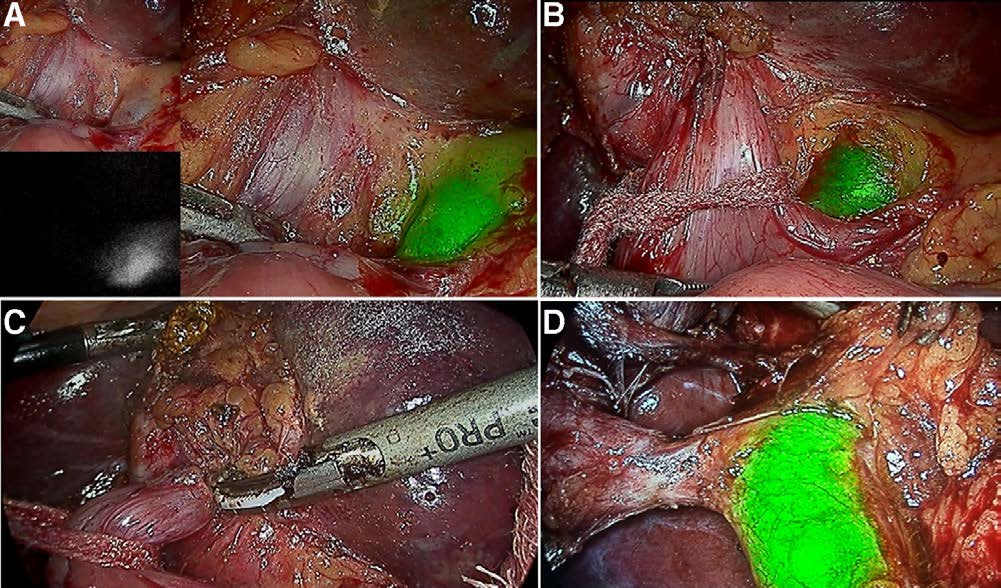
(a) ICGF showing CBD location in an obscure hepatocystic triangle with a choledochoduodenal fistula; pure laparoscopic, NIRF and NIRFA images in the left upper corner, left lower corner and right side respectively; (b) Cholecystoduodenal fistula dissection and looping aided by real-time NIRFA-CBD; (c) Cholecystoduodenal fistula takedown in progress; (d) ICGF demonstrating critical view of safety after fistula takedown. CBD = common bile duct, ICGF = ICG fluorescence, NIRF = near-infrared fluorescence, NIRFA = near infrared fluorescence augmented.

ICGF findings are shown in Table 1. CBD and cystic duct (CD) visualization with ICGF was seen in 67(98.5%) and 62(91%) patients respectively. The CBD and CD visualization was in 32 and 30 patients, respectively in UGS group. This was 35 and 30 patients, respectively in the CGS group. CVS was achieved in 61 patients (Fig. 2). In the remaining 7 patients, fundus first gallbladder mobilization was performed and ICGF guided LC and subtotal cholecystectomy with a small remnant in 5 and 2 patients, respectively. Significant bleeding was not encountered in any patient. The presence of ooze secondary to inflammation in patients with acute cholecystitis did not obscure the NIRFA including a single patient with a spurter from the cystic artery adherent to cystic duct (Fig. 3). The single patient with symptomatic remnant gallbladder calculi also had a cholecystoduodenal fistula. ICGF in this patient helped early identification of CBD that helped in safe looping and takedown of the fistula (Fig. 4).

**Table 1:** Details of Gallstone disease, CVS and visualization of cystic duct and CBD on NIRF.

	n	CVS		NIRF CD not seen	NIRF CBD not seen
		A	NA		
UGS	32	32	0	2	0
AC	19	17	2	4	1
AC with CCF	1	1	0	0	0
AC with CBDS	4	4	0	0	0
CBDS	4	4	0	0	0
CBDS with CDDF	1	1	0	0	0
CBDS with CDF	1	1	0	0	0
GSP	3	3	0	0	0
GSP with CBDS	2	2	0	0	0
RGB with CDF	1	1	0	0	0
Total	68	66	2	6	1

A = achieved, AC = acute cholecystitis, CBDS = CBD stones, CCF = cholecystocolonic fistula, CDDF = choledochoduodenal fistula, CDF = cholecystoduodenal fistula, CVS = critical view of safety, GSP = gallstone pancreatitis, NA = not achieved, NIRF = near infrared fluorescence, UGS = uncomplicated gallstone disease, RGB = remnant gallbladder.

### 3.1. Intraoperative cholangiogram (n = 18)

While IOC was attempted in 18 patients, it was successful in 17 and cystic duct cannulation failed in one with CBD stone. IOC was successful, overall in 18 patients (26.4%), including 2 (6.25%) and 16 (44.4%) patients in the UGS and CGS groups respectively. The indications for IOC were GSP (5), altered LFT (4), prior to laparoscopic CBD exploration (6) and cystic duct stones/sludge during surgery (2). IOC helped in identification of CBD stones additionally in 2 patients, one with gall stone pancreatitis and another with altered LFT additionally. In the single patient despite a failed cannulation of cystic duct (vide supra), a LCBDE was performed making up a total of 7 patients who underwent LCBDE.

### 3.2. CBD stone management (n = 12)

**Figure 5.**
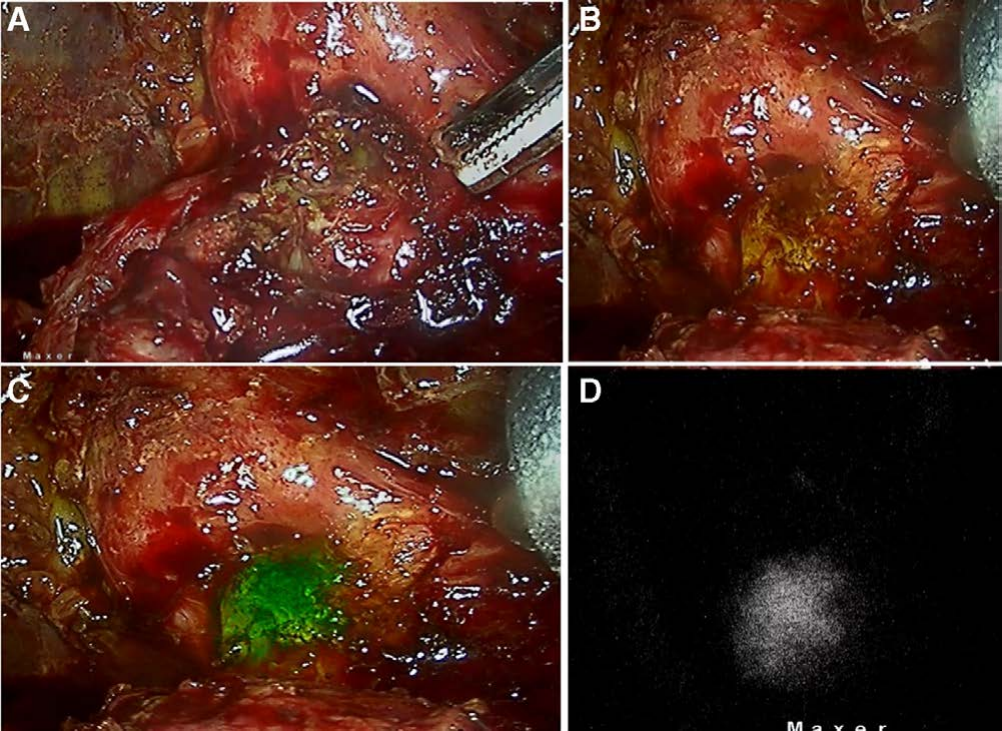
In a patient undergoing Laparoscopic CBD exploration, (a) Pure laparoscopic view during dissection of difficult hepatocystic triangle; (b) ICGF demonstrating cystic duct and CBD junction and the cleared supraduodenal CBD in preparation for choledochotomy; (c) Supraduodenal choledochotomy showing fluorescent bile exiting the choledochotomy; (d) Trans-cystic duct instillation of ICG demonstrating no fluorescent leak from choledochotomy closure site. CBD = common bile duct, ICGF = ICG fluorescence.

Details of management of CBD stones are shown in Table 2. All patients had pain at presentation. Four of them had associated acute cholecystitis; Tokyo grade I in 3 and grade III in one. Two of the former patients had an ERC and LC and one of them a LC and laparoscopic CBD exploration. The latter patient had a percutaneous cholecystostomy and subsequently a LC and laparoscopic CBD exploration. NIRFA-CBD and NIRFA-CD were seen in all patients including the single patient with a CBD stent in situ, placed during preoperative ERC following CBD clearance. NIRFA guided exposure of CBD, choledochotomy and after the suture closure of choledochotomy, the transcystic duct instillation of ICG confirmed absence of leak (Fig. 5).

**Table 2:** Details of management in patients with CBD stones.

Case no	IOC	Name of the procedure	Remarks
1	Y	LC + LCBDE	
2	Y	LC + LCBDE	
3	Y	LC + LCBDE	Detected on IOC
4	Y	LC + LCBDE	
5	Y	LC + LCBDE	
6	Y	LC + Intra-op ERCP	Detected on IOC
7	Y	LC	PRO-ERCP, CBD clearance and CBD stenting
8	F	LC	POO-ERCP, CBD clearance and CBD stenting
9	Y	LC	No CBD calculi on IOC
10	Y	LC + LCBDE	
11	Y	LC + LCBDE	
12	F	LC + LCBDE	

F = failed, IOC = intraoperative cholangiogram, LC = laparoscopic cholecystectomy, LCBDE = laparoscopic CBD exploration, POO-ERCP = postoperative ERCP, PRO-ERCP = preoperative ERCP, Y = yes.

### 3.3. CD stones (n = 2)

Cystic duct stones were retrieved through cystic ductotomy in 2 patients. Flourescent leak became evident at ductotomy in both when the stones were retrieved. The CD was dealt subsequently by clipping in the usual manner.

### 3.4. Bilioenteric fistulae (n = 4)

**Figure 6.**
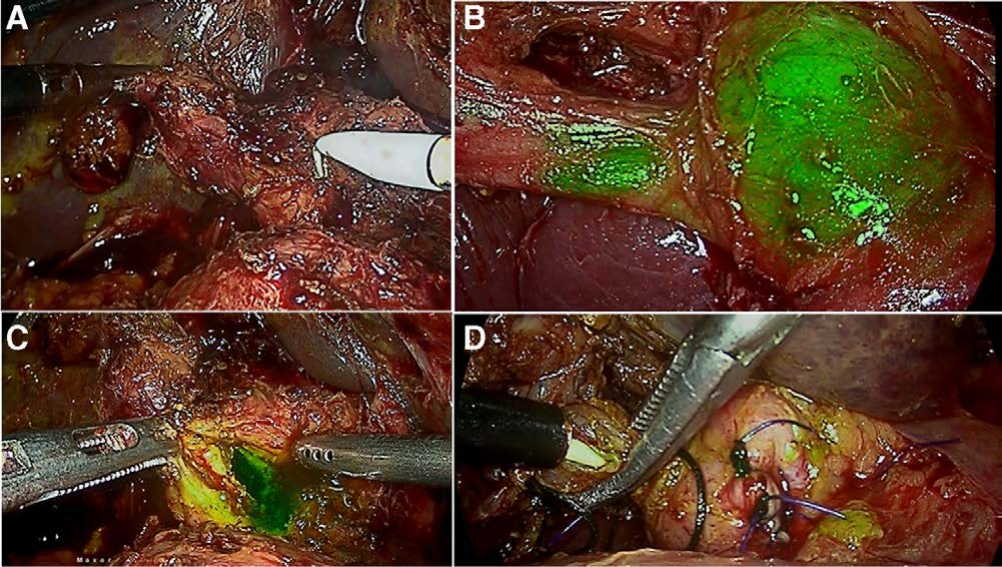
(a) Pure laparoscopic image showing choledochoduodenal fistula; (b) Bile leak from CBD side of fistula after its takedown which is not clearly visible; (c) Fluorescent bile leak clearly visible in the corresponding NIRFA image; (d) The same leak in a NIRF image. CBD = common bile duct, NIRFA = near infrared fluorescence augmented.

Three patients with CBD stones had an internal fistula; cholecystoduodenal, cholecystocolonic and choledochoduodenal fistula in one patient each (Fig. 6). One patient undergoing completion cholecystectomy for remnant gallstones had a chlecystoduodenal fistula.

### 3.5. Postoperative outcome

There was no conversion to open surgery and there were no CBD injuries. Three patients (4.4%) had morbidity (Clavien-Dindo Grade 1); mild acute pancreatitis in 2 and postoperative ileus in one which recovered with conservative measures. All other patients had an uneventful postoperative course. The median postoperative stay was 1 (range 1–5) day. The CBD stents in eleven patients were removed 3 weeks later at their follow-up outpatient visits.

## 4. Discussion

ICGF during LC is widely reported in the last few years to enable a safe cholecystectomy. Visualizing the extrahepatic biliary tree (CBD and CD fluorescence) to increase the safety of LC is the purported benefit of ICGF. An ability to achieve a CVS with greater confidence and faster time to achieve it has been reported in a recent randomized trial.^[9]^ In a recent comparative study, among patients undergoing LC at a large academic center, without (no ICGF group) and with (ICGF group) the use of ICGF, the authors showed a significantly faster time in achieving CVS, lesser time in completing the cholecystectomy, quicker discharge from hospital and no major bile duct injuries in the latter group.^[10]^ The recent Shanghai international consensus statement on hepato-biliary fluorescence recommended its routine use to increase safety during LC.^[2]^

This relatively new technology in laparoscopic surgery is, however, limited to a few centers. Interpretation of the currently available data and its application by general surgeons in clinical practice becomes difficult, given the wide spectrum of clinical presentation seen in patients with gall stone disease. Additionally, the ICG related technology for laparoscopy has been evolving continuously towards providing a real-time augmentation of images to provide greater comfort to the surgeon during conduct of operations. The NIR imaging system that was used in this study had several unique features that provided high resolution images with true depth perception that enabled excellent visualization of extra-hepatic biliary tree during LC for CGS. First, it had 4 separate complementary metal-oxide-semiconductor chips. The dedicated NIR source apart from the red, blue and green sources allowed for high resolution images. Second, the system allowed 3 images, namely; monochrome (dedicated NIR image), augmented image and white light image in 1 frame (as a “picture in picture”[PIP]) without the need to swtich from laparoscopic image to NIR image and back to laparoscopic image. Third, the 4 chip system with a dedicated NIR source allowed for an independent shutter speed adjustment of NIR source to increase or decrease the sensitivity of NIR images. Finally, the system was intuitive to suppress background liver noise. This is a significant improvement over other available systems which utilize 2 or 3 chips that do not have the above-mentioned advantages. Also, the need to switch back and forth between white light and NIR images interferes with the surgical flow.

Dosing and timing of ICG prior to LC has been controversial. Ishizawa et al, reported optimum visualization of extra-hepatic biliary tree with 2.5 mg of ICG given thirty minutes prior to the procedure.^[11]^ All patients in this study received 2.5 mg of ICG given 2 hours before surgery that enabled optimum visualization of extrahepatic biliary tree. Patients with CGS undergoing LC have a higher risk of conversion of the laparoscopic procedure to an open operation and a higher risk of bile duct injury.^[12]^ The role of ICGF in a spectrum of complicated gall stone disease, utilizing a state of the art laparoscopic NIRF imaging system with a “four chip” camera, is discussed below.

### 4.1. ICGF during LC for CGS

In our study, NIR fluorescence of CBD was seen in 98.5% of all the patients. It was seen in 100% UGS versus 98.5% in CGS. CD visualization was not possible in 4 patients and all belonged to the acute cholecystitis subgroup. The difference in CBD and CD visualization between the acute cholecystitis and non-acute cholecystitis group were not statistically significant (Table 3). CBD visualization with ICGF in the complications other than acute cholecystitis are similar to that of UGS. The small numbers in the various subgroups of CGS did not permit any meaningful statistical analysis. It is important to note that, whereas “CBD seen on ICGF” was a goal achieved at the beginning of the procedure in most patients, in some, this was possible only after initial dissection of the hepatocystic triangle. This “NIR blind period” in the latter group of patients emphasizes the need to adhere to standard dissection techniques to avoid a bile duct injury. The combination of cystic duct visualization along with CBD visualization have been shown to enhance the safety of achieving a CVS. In summary, while achieving CVS is an established benchmark for avoiding a bile duct injury, CBD visualized on ICGF, provides a visual aid in achieving it. Additionally, in patients where CVS cannot be achieved, ICGF aids in completion of the cholecystectomy (see below).

**Table 3:** Comparison of different groups with respect to non-visualization of CD and CBD on ICGF during laparoscopic cholecystectomy.

	No	NIR-CD not seen	NIR-CBD not seen
AC	24	4	1
Non-AC	12	0	0
Complication			
- One	26	4	1
- More than one	10	0	0

AC = acute cholecystitis, CBD = common bile duct, CD = cystic duct.

### 4.2. ICGF in Acute cholecystitis

**Figure 7.**
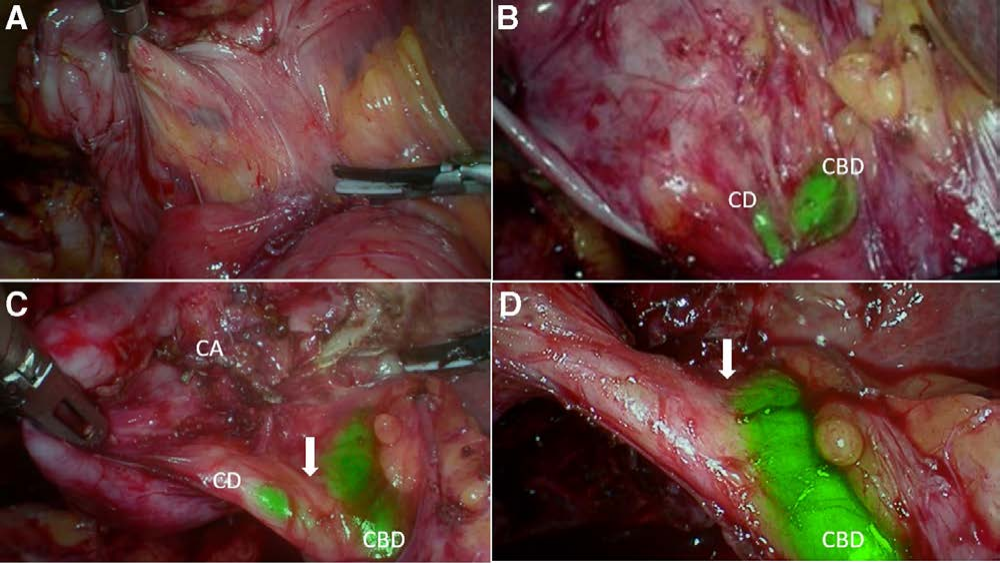
(a) Pure laparoscopic image showing obscure hepatocystic triangle; (b) ICGF showing location of cystic duct and CBD: (c) ICGF after further dissection showing cystic artery, cystic duct and CBD, arrow showing cystic duct-CBD junction; (d) ICGF demonstrating cystic duct, CBD and common hepatic duct prior to clipping of cystic duct, arrow showing cystic duct-CBD junction. CBD = common bile duct, ICGF = ICG fluorescence.

Safe principles for dissection during LC including demonstration of CVS are aimed at avoiding major bile duct injuries. The above stated goals remain unchanged with the use of ICGF, but the visual aid it provides to locate the bile duct, can only enhance the ability and safety of dissection (Fig. 7). ICGF would be useful in patients with acute cholecystits where a difficult dissection would be expected. However, edematous and thickened tissues may interfere in visualizing CBD in some these patients. Hiwatashi et al reported non-visualization of CD and CBD in 6.2% of their patients. Additional 6 patients had non-visualization of CD alone. All patients with non-visualization belonged to the acute cholecystitis subgroup in their study. They reported a correlation of inflammatory thickening of tissues with non-visualization of EHBT.^[6]^ In 23 of 24 patients with acute cholecystitis, visualization of CBD with ICGF was possible. The highly sensitive NIR images in the present study are a consequence of dedicated NIR source with an intuitive ability to reduce the shutter speed that enabled better visualization of the extrahepatic biliary tree. An obscure anatomy found during LC for acute cholecystitis is among the leading cause for a subtotal cholecystectomy. There are currently few data on rates of subtotal cholecystectomy with the use of ICGF during LC. In our study, 2 patients in the AC subgroup of CGS group, where CVS could not be achieved had a subtotal cholecystectomy. In our study, the CD non-visualization was 6.25% versus 11%, and rate of subtotal cholecystectomy was 0% versus 5.5% in patients with UGS and CGS respectively. It appears that STC is more likely in patients with CD non-visualization on ICGF (2 out of 6 in this study). As a corollary, subtotal cholecystectomy is less likely in patients with CD visualization (zero out of 62). However, CVS was possible in 4 of the 6 patients with non-visualization of CD and it avoided a subtotal cholecystectomy in them. Also, in the 2 patients that underwent a subtotal cholecystectomy, ICGF of CBD provided guidance to ensure a very small remnant which is an important measure to avoid future symptoms in the remnant gallbladder. We have found NIRF images to provide useful information with regards to visualization of the extra-hepatic biliary tree early in dissection when augmented images (NIRFA) alone may not provide the desired information considering the lesser sensitivity of NIRFA compared to NIRF alone. This underscores the need to critically assess the NIRF images, particularly in the presence of inflammation and thickened tissues.

### 4.3. ICGF in choledocholithiasis

Wang et al, recently reported on utility of ICGF in detection of CBD during difficult CBD exploration in 2 patients.^[13]^ Our early experience with ICGF in 8 patients undergoing LCBDE showed the following advantages. Firstly, it increased the ease of exposure of supraduodenal CBD. Secondly, there was no need for aspiration of bile to confirm the CBD (Fig. 5). Thirdly, the visualization of the CD-CBD junction in all ensured that the choledochotomy was done in CBD and not CHD. Fourthly, the bleeding from the edge was easily managed with bipolar coagulation as bleeding point was clearly visible on the real time NIRFA while it was unclear in the pure laparoscopic view. Finally, instilling ICG through the cystic duct at the completion of procedure confirmed a secure closure of choledochotomy when there was no leak of fluorescent fluid from the closed choledochotomy site. This is a definite advantage over use of a colored dye such as methylene blue which stains the operative field. We believe that with increasing familiarity with ICGF during LC, there is a possibility of its increased utilization for LCBE among practicing surgeons.

### 4.4. ICGF in special situations

#### 4.4.1. Remnant GB.

Remnant gall bladder is the result of a subtotal cholecystectomy done during previous surgery. The most common etiology reported for a subtotal cholecystectomy is an obscure Calot’s region.^[14]^ A large series reported, a 10% incidence of internal fistula and the need for laparoscopic to open conversion for remnant cholecystectomy in 39.5%.^[15]^ Utsunomiya, reported use of ICGF in 2 patients undergoing completion cholecystectomy for symptomatic remnant gallbladder stones. In their report, both patients were given intravenous ICG the previous day. While it showed the CBD in first enabling successful laparoscopic completion of the procedure. Misinterpretation of anatomy led to a duodenal perforation and they cautioned against heavy reliance of ICGF alone.^[16]^ The operative images provided in their report show significant background “noise” from liver and other structures around CBD, which was not the case in our experience. We had used ICGF in 1 patient undergoing a remnant cholecystectomy. During initial dissection, a CDF was suspected. We could visualize the EHBT on NIRF images when the NIRFA images were unclear (vide supra). ICGF helped visualizing the CBD during the dissection, looping of the fistula and its takedown. Subsequently the CBD was clearly visualized, that guided achieving CVS and successful completion of the procedure. These are obviously difficult procedures and ICGF is an important tool in avoiding a possible conversion to an open procedure.

#### 4.4.2. Bilio-enteric fistula.

Cholecystoenteric fistula are usually detected intraoperatively and result in conversion to an open procedure in 20% of patients to manage the fistula and complete the cholecystectomy.^[17]^ There were 4 patients with bilio-enteric fistula in this study. Despite the hepatocystic region that was obscured by the fistula, the CBD could be seen on ICGF prior to dissection of the fistula in all 4 patients. In 1 patient undergoing remnant cholecystectomy, where the augmented images (NIRFA) were unclear earlier in the course of surgery, pure NIRF images helped in localization of extrahepatic biliary tree (Fig. 1c,d,e). Thus, ICGF helped in careful dissection, looping and takedown of the fistula (Fig. 4). Further to fistula takedown, ICGF showed CBD and cystic duct fluorescence in all 4, on the augmented images (NIRFA) in real-time. While the higher sensitivity of NIRF images allows for assessing the CBD location in these difficult situations prior to fistula takedown, NIRF and NIRFA images complement each other during the subsequent dissection. Although dense adhesions were encountered CVS could be achieved in all 4 with the aid of ICGF.

#### 4.4.3. CD issues (CD stones).

In a large series of patients undergoing cholecystectomy, the incidence of cystic duct remnant calculus was 4.19%, and 0.02% among patients who underwent STC and conventional LC, respectively.^[18]^ Several strategies have been suggested to decrease the problem of remnant CD stones during LC, viz; milking the stones felt in the CD towards the gall bladder before clipping, dissecting the CD upto one centimeter from the CBD and the routine use of IOC.^[19,20]^ The present study findings suggest interesting applications that need further prospective evaluation. First, non-visualization of cystic duct may indicate a cystic duct block by a stone. A cystic ductotomy after clipping the gall bladder side of CD, with a milking maneuver, retrieved cystic duct calculi that was followed by a free flow of fluorescent bile in 2 patients in this study (Fig. 2d). This finding may indicate a CD free of stones. Second, dissecting CD upto 1 cm from its junction with CBD would carry a risk of CBD injury when the CD-CBD junction is not apparent during routine LC. Since CD-CBD junction was visualized on ICGF in nearly 91% of our patients and this might aid in leaving a smaller remnant of CD. Third, when an IOC is performed, on occasion, it is unclear whether the mucosa is cut to allow for cannulation and enlarging the CD ductotomy risks its complete transection. Bile leak at ductotomy site is obvious during ICGF by the leak of fluorescent bile indicating mucosal entry and thereby helps avoiding an unduly large ductotomy. Finally, after completion of LC, a careful inspection of cystic duct site, may detect a bile leak with ICGF.

#### 4.4.4. IOC and ICGF.

IOC can be performed only after the dissection has progressed to an extent where cystic duct has been dissected to facilitate cannulation. In patients with CGS, we found ICGF helped us to visualize the extra-hepatic biliary tree even before the cystic duct was dissected and had the additional advantage of a more continuous guidance when compared to IOC where repeating the investigation could be cumbersome. While ICGF provided details pertaining to biliary anatomy during cholecystectomy, IOC played a distinct role in evaluation and dealing with associated pathology such as common bile duct stones in this study. Their roles therefore need to be seen as complimentary in managing patients with CGS. A recent meta-analysis by Lim et al, showed better visualization of extra-hepatic biliary tree with ICGF in comparison to IOC.^[21]^

The limitations of current study are its retrospective nature, small series of CGS and the inherent referral bias to an advanced laparoscopy center. Nevertheless, ICGF during LC offers several potential advantages in patients with CGS. Unlike its role in UGS that is primarily to guide the surgical dissection to identify target structures, in CGS disease it has several other potential roles that need further prospective study. It is to be noted that the highly sensitive NIRF imaging system with 4 chip camera used in the present study and the results thereof may not be generalizable for all patients undergoing NIRF guided surgery considering the diverse nature of available NIRF camera systems in the market. Although our study was not designed to evaluate the cost effectiveness of the technology, the relatively low cost of ICG was not prohibitive for routine use. The rarity of some of the disease presentations of CGS can be addressed only when prospective studies using ICGF during LC do not exclude such presentations. Thus, large multi-center prospective studies that are inclusive of patients with all complications of gallstones would help us understand its potential roles beyond the regular application during LC alone.

## 5. Conclusions

In our small series of patients with CGS, ICGF guidance enabled a LC and laparoscopic subtotal cholecystectomy in 94% and 6% of patients respectively. The study highlights potential roles and advantages with ICGF guided laparoscopic management for CBD stones, bilioenteric fistula, completion cholecystectomy and cystic duct stones. Large scale multicenter prospective studies are required to clarify the role of ICGF in the wide spectrum of CGS.

## Acknowledgments

We would like to thank Prof VK Kapoor, Prof and Head of HPB Surgery, Mahatma Gandhi Medical College, Jaipur, India for his valuable suggestions. Authors would like to acknowledge, Dr Mahesh V—Biostatistician, Associate Professor, Department of community medicine, CIMS, Chamarajnagar, Karnataka, India for his help in statistical analysis.

## Author contributions

Both authors confirm their contribution in the preparation, review and are in agreement with the final manuscript being submitted. (Details in CRediT format below)

**Conceptualization:** Srikanth Gadiyaram.

**Data curation:** Ravi Kiran Thota.

**Formal analysis:** Ravi Kiran Thota, Srikanth Gadiyaram.

**Methodology:** Srikanth Gadiyaram.

**Software:** Ravi Kiran Thota, Srikanth Gadiyaram.

**Validation:** Srikanth Gadiyaram, Ravi Kiran Thota.

**Visualization:** Srikanth Gadiyaram.

**Writing – original draft:** Srikanth Gadiyaram, Ravi Kiran Thota.

**Writing – review and editing:** Srikanth Gadiyaram, Ravi Kiran Thota.
